# Exploring the Interplay Between Emotions and Medical Decision-Making: Insights from an Empirical Patient-Centred Study

**DOI:** 10.3390/healthcare14172835

**Published:** 2026-09-03

**Authors:** Nan Ji, Yong Liu, Ying Wang

**Affiliations:** 1 School of Design & Art, Wuxi University of Technology, Wuxi 214000, China; 2 School of Design, Jiangnan University, Wuxi 214000, China; 3 Department of Orthopaedics, Wuxi Hospital of Traditional Chinese Medicine, Wuxi 214000, China; liuyong301@outlook.com (Y.L.); wy87312@126.com (Y.W.)

**Keywords:** patient-centred medical decision-making, emotions in healthcare, anxiety and hopefulness, CB-SEM, patient involvement, decision satisfaction and regret

## Abstract

Background: Patient-centred decision-making is shaped by clinical information as well as patients’ emotional and relational experiences during care. This cross-sectional study examined how positive and negative emotions, physician trust, perceived empathy, and patient involvement were associated with patient-reported decision outcomes. Methods: A structured 30-item questionnaire using 5-point Likert scales was administered to 600 adult patients recruited by convenience sampling from Wuxi Hospital of Traditional Chinese Medicine, Jiangsu Province, China. The measurement model assessed negative emotions, positive emotions, trust in physicians, physician empathy, patient involvement, and decision outcomes. Reliability, exploratory factor analysis, confirmatory factor analysis, common-method-bias diagnostics, and covariance-based structural equation modelling were conducted. Results: The model showed acceptable-to-good fit (CFI = 0.961, TLI = 0.954, RMSEA = 0.043, SRMR = 0.038). Positive emotions (β = 0.312), physician trust (β = 0.398), and physician empathy (β = 0.361) were positively associated with patient involvement, whereas negative emotions were negatively associated with involvement (β = −0.284; all *p* < 0.001). Patient involvement partly mediated associations between emotional/relational factors and decision outcomes and also moderated predictor-outcome associations. Conclusions: In this single-institution sample, emotional states and relational perceptions were meaningfully associated with patient involvement and decision outcomes. Given the cross-sectional design, these findings indicate associations rather than causal effects; longitudinal and multi-centre studies are needed before causal or broadly generalizable conclusions can be drawn.

## 1. Introduction

For a long time, the process of making medical decisions has been predominantly seen as combining rationality, rule-based approaches and professional judgement [1]. However, the recent literature has increasingly emphasized that patients rarely make decisions based solely on cognitive reasoning [2]. Emotions such as anxiety, fear, hope, and trust play a central role in shaping how patients understand medical information, weigh risks and benefits, and ultimately make decisions [3]. The emotional aspect is extremely important in the context of patient-centred care, in which the patient’s values, preferences and personal experiences are merged with the clinical process [4]. Interpreting the relationship between emotions and decision-making is thus a prerequisite for both theory and practice in the healthcare sector to make progress [5].

The doctor–patient relationship provides an important clinical context in which emotions may shape medical decisions [6]. Trust in the doctor, perceived empathy, and the quality of communication can either dampen or heighten patients’ emotional responses during the consultations [7]. To illustrate, empathy-laden communication can diminish decisional conflict and promote trust, whereas distrust may increase anxiety and result in the refusal or postponement of the recommended treatments [8]. Even though these dynamics are recognized, there is still a lack of empirical research that measures the role of emotions in decision-making [9]. Most existing research relies on conceptual frameworks or small-scale qualitative evidence; therefore, large-scale quantitative validation remains limited.

The present study addresses this gap by quantitatively examining how emotional and relational factors are associated with patient-centred medical decision-making [10]. This study examines emotional factors, trust, and empathy as predictors of patient involvement and decision outcomes, including acceptance, satisfaction, and regret [11]. Using a relatively large sample and structural modelling, this study moves beyond descriptive analysis by examining hypothesized associations among emotional and relational constructs [12]. In this way, it provides an integrated framework for examining the psychological basis of medical decision-making [13].

A contribution of this study is the integration of emotional and relational variables within a unified empirical model of patient-centred medical decision-making. Building on previous research, this study explicitly examines emotions as important factors associated with patient involvement and decision outcomes. The findings are expected to provide practical guidance for physicians by demonstrating that patients’ emotional needs should be valued as highly as clinical information. These findings may support a more patient-centred understanding of medical decision-making by suggesting that emotions should be considered alongside clinical information and patient preferences.

### 1.1. Research Questions and Hypothesis Development

**RQ1.**  *What is the relationship between patients’ emotional states (positive and negative emotions) and their involvement in medical decision-making?*

**H1.**  *Patients’ emotional states (positive and negative emotions) are significantly associated with their level of involvement in medical decision-making*.

Prior studies in health psychology suggest that emotions are closely related to cognitive processing and engagement in medical decisions. Negative emotions such as anxiety and fear may be associated with less active participation, while positive emotions such as hope and confidence are linked to greater involvement. This aligns with dual-process theories of decision-making, which emphasize the role of affective states in shaping patient behaviour.

**RQ2.**  *How are physician trust and empathy associated with patient involvement in the medical decision-making process?*

**H2.**  *Physician trust and empathy are positively associated with patient involvement in the medical decision-making process*.

Trust in the physician and perceived empathy are central to patient-centred care. Literature on shared decision-making shows that when patients believe their physician is acting in their best interest and demonstrates empathy, they are more likely to engage actively in discussions and express preferences. This relationship is consistent with models of therapeutic alliance and communication in healthcare.

**RQ3.**  *In what ways are patient emotions, trust, and empathy associated with decision outcomes such as confidence, satisfaction, and regret?*

**H3.**  *Patient emotions, trust, and empathy are significantly associated with decision outcomes such as confidence, satisfaction, and regret*.

Decision outcomes are not only related to rational evaluation of risks and benefits but also to emotional and relational factors. Positive emotions, high trust, and physician empathy are often associated with greater confidence and satisfaction, while negative emotions and low trust are associated with higher regret. This is supported by empirical findings in patient satisfaction research and decision regret studies.

**RQ4.**  *How does patient involvement shape the associations between emotions, trust, empathy, and medical decision outcomes?*

**H4.**  *Patient involvement moderates the relationship between emotions and medical decision outcomes, such that higher involvement is associated with stronger favourable associations and weaker unfavourable associations*.

Active involvement in decision-making empowers patients to align choices with their values, which can buffer the unfavourable association of anxiety or fear and amplify the benefits of hope and confidence. Moderation by involvement is consistent with empowerment theory and shared decision-making frameworks, which highlight the protective role of patient participation in achieving favourable outcomes.

In the present model, patient involvement was specified in two complementary but analytically distinct ways. As a mediator, involvement represents a decision-process mechanism through which emotional and relational experiences are statistically linked with patient-reported outcomes. As a moderator, involvement represents a boundary condition that tests whether the strength of emotion-outcome and relationship-outcome associations differs across levels of participation. Because the data are cross-sectional, these specifications are interpreted as theoretically guided associations rather than temporal causal pathways.

**RQ5.**  *To what extent are emotions, trust, and empathy collectively associated with patient satisfaction and willingness to recommend the physician?*

**H5.**  *Emotions, trust, and empathy collectively explain a significant proportion of variance in patient satisfaction and willingness to recommend the physician*.

Patient satisfaction is multidimensional, influenced by emotional experiences, relational trust, and perceived empathy. These constructs jointly shape overall evaluations of care and future behavioural intentions, such as recommending the physician. This integrated association is supported by patient experience models, which emphasize the interplay of affective and relational factors in shaping satisfaction.

### 1.2. Objectives

The objectives of this study were to identify the key emotional, relational, and involvement-related constructs that are associated with medical decision-making, including anxiety, hope, confidence, trust, empathy, and patient involvement. This study also aimed to use validated measurement instruments and CB-SEM modelling to examine the hypothesized pathways linking emotions and relational factors with patient-centred outcomes. In addition, the study sought to distinguish the direct, indirect, and moderated associations of emotions, trust, and empathy with patient involvement and decision outcomes, with particular attention to mediation and moderation processes. Overall, this study aimed to integrate emotional and relational variables into a unified CB-SEM framework and generate evidence-based insights for improving patient-centred clinical decision-making.

### 1.3. Organization of the Paper

This paper is divided into six major parts to critically link the concept development phase with evidence-based validation. Section 1 provides the introduction, outlining the importance of emotions and relational constructs in medical decision-making, the research gap, and hypotheses. Section 2 reviews related literature on emotions, trust, empathy, involvement, and decision outcomes, highlighting limitations of prior studies. Section 3 describes the materials and methods, including research design, data collection, variables, and CB-SEM analysis. Section 4 presents the data analysis, covering reliability, factor analyses, mediation, moderation, and model fit indices. Section 5 reports the results, detailing descriptive statistics, hypothesis testing, and structural model findings. Finally, Section 6 offers the conclusion, interpreting results in light of existing literature, emphasizing theoretical and practical contributions, and suggesting directions for future research.

## 2. Related Works

The emotional aspects that are part of the patients’ decisions have mostly been disregarded in the previous research on medical decision-making, which has heavily relied on rational and clinical frameworks. While recent studies are already drawing attention to the physician-patient relationship and trust as major components, the occurrence of empirical evidence regarding emotions is still scarce.

Broadbridge and Venetis (2024) [14] examined how pre-encounter anxiety and hope shape risk appraisal in outpatient consultations using a cross-sectional survey with validated affect scales; they found anxiety elevated perceived risk and reduced recommendation acceptance, but self-report and single-timepoint measurement limited causal inference. Pel-Littel et al. (2024) [15] adapted language anxiety constructs to medical decision contexts, using a structured questionnaire to test whether situational anxiety correlates with decisional conflict; they reported moderate correlations, yet common-method bias and absence of longitudinal follow-up reduced interpretability of temporal effects.

Chen et al. (2020) [16] quantified the association between physician trust and acceptance of recommended procedures in cardiology clinics through multivariate regression; higher trust predicted acceptance after adjustment, though single-specialty sampling and omitted clinic-level clustering threatened external validity. Campos et al. (2024) [17] used the CARE Measure in internal medicine to test whether perceived empathy relates to decisional conflict and satisfaction; empathy was inversely associated with conflict and positively with satisfaction, but the cross-sectional design precluded estimation of downstream adherence or regret.

Goto et al. (2021) [18] analyzed patient involvement via SDM-Q-9 in primary care and linked higher involvement to lower stress and greater confidence; results highlighted process benefits, yet time pressure and workload were not measured, limiting conclusions about feasibility in routine practice. Bradley et al. (2021) [19] conducted a longitudinal study of prostate cancer patients, measuring baseline emotions and six-month regret with a validated scale; negative affect at decision predicted later regret, but disease-specific sampling and attrition constrained generalizability and statistical power.

Netemeyer et al. (2020) [20] assessed the effect of wellbeing knowledge and proficiency on decision quality, finding that low literacy amplified anxiety’s association with conflict; although the study improved confounding control, brief screening tools may have misclassified literacy levels, introducing measurement error. Chen et al. (2023) [21] evaluated a 0–100 visual analogue risk scale during imaging decisions, showing that higher perceived risk reduced acceptance of non-urgent procedures; immediate post-encounter assessment improved relevance, but lack of calibration against objective risk weakened interpretability.

Wu et al. (2022) [22] tested a mediation model where perceived empathy increases trust, which then predicts acceptance, using path analysis with outpatient data; indirect effects were significant, yet treating ordinal Likert items as continuous and ignoring clinic nesting may have biased estimates. Zhang et al. (2024) [23] modelled decisional conflict as a mediator between anxiety and final decision using structural models; mediation was supported, though cross-sectional timing and shared method variance limited claims about directionality and true indirect effects.

Bollegala et al. (2022) [24] surveyed multispecialty clinics to examine whether perceived emotional support predicts adherence; emotionally supported patients reported higher adherence, but reliance on self-reports and non-probability sampling introduced social desirability and selection biases. Dell’Osso et al. (2023) [25] applied covariance-based SEM to integrate emotions, empathy, trust, and acceptance across 600 outpatients, reporting acceptable fit and significant paths; nevertheless, mixed categorical/continuous outcomes without robust estimators and limited measurement invariance testing constrained confirmatory strength.

### Conceptual Research Model

A Conceptual Research Model is a pictorial representation that illustrates the variables and how they were assumed to be related in a study. It assists the researcher to clearly demonstrate the way in which independent, mediating, and dependent constructs are supposed to interact.

**Figure 1:**
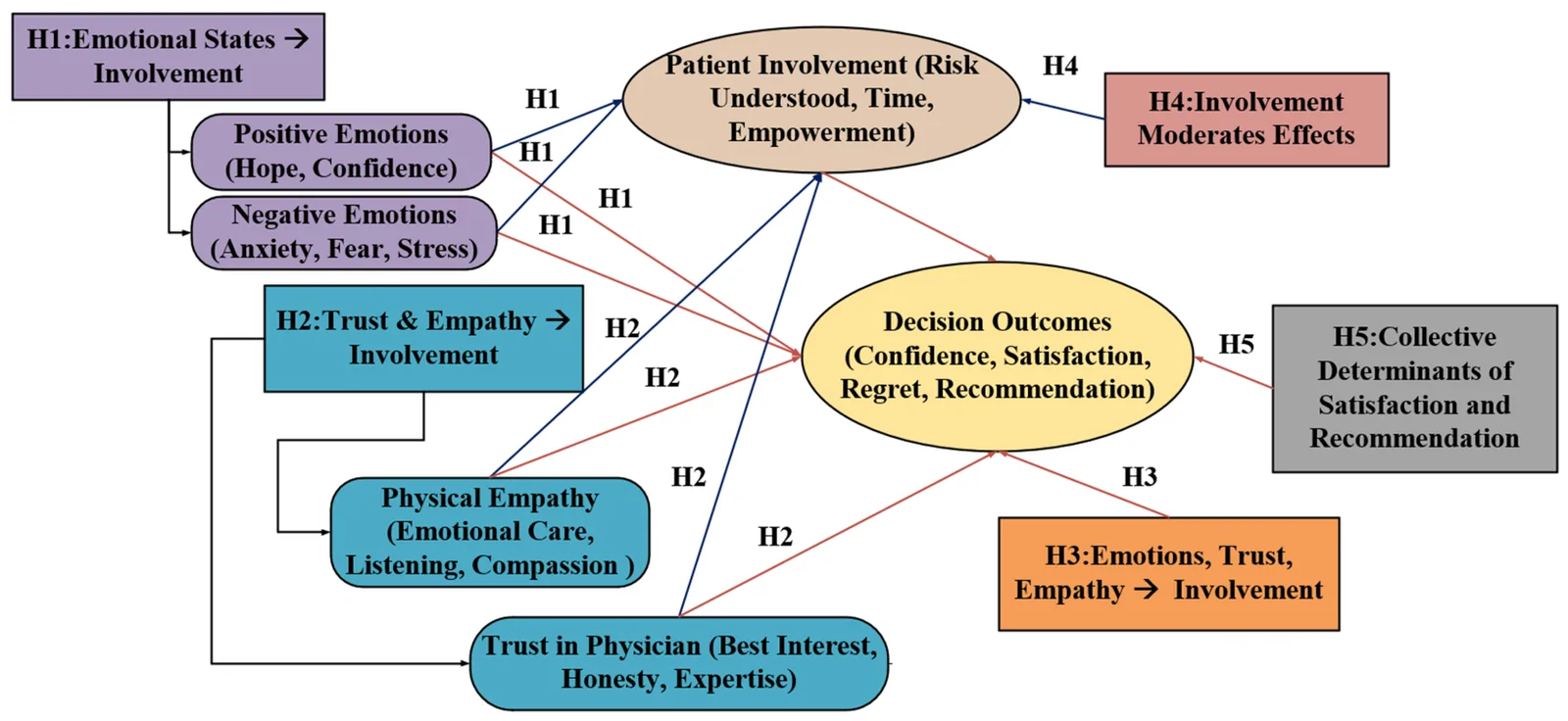
Conceptual Framework Linking Emotional States, Relational Factors, Patient Involvement, and Decision Outcomes.

Figure 1 outlines the conceptual framework of this study, depicting the hypothesized relationships among emotional states, trust, empathy, patient involvement, and decision outcomes. As shown, H1 proposes that positive emotions (hope, confidence) are associated with higher involvement, while negative emotions (anxiety, fear, stress) are associated with lower involvement. H2 highlights the role of physician trust and empathy, suggesting they are positively associated with patient involvement. H3 extends these constructs to decision outcomes, indicating that emotions, trust, and empathy are significantly associated with confidence, satisfaction, and regret. H4 positions patient involvement as a moderator of these associations. Finally, H5 integrates these pathways by examining the variance in satisfaction and willingness to recommend the physician explained by emotions, trust, and empathy.

## 3. Materials and Methods

The primary aim of this study was to investigate the associations of emotions with medical decision-making from a patient-centred perspective. A detailed questionnaire encompassing six major aspects was developed: emotional states, trust in physicians, perceived empathy, patient participation, decision outcomes, and patient-centred experiences. In an effort to ensure that there was a comprehensive outlook, the information was gathered from a diverse group of patients in varying health settings. The entire research process was conducted under ethical oversight. This research placed strong emphasis on systematically recording patient voices and experiences, thereby enabling exploration of the emotional dimensions of medical choices.

**Figure 2:**
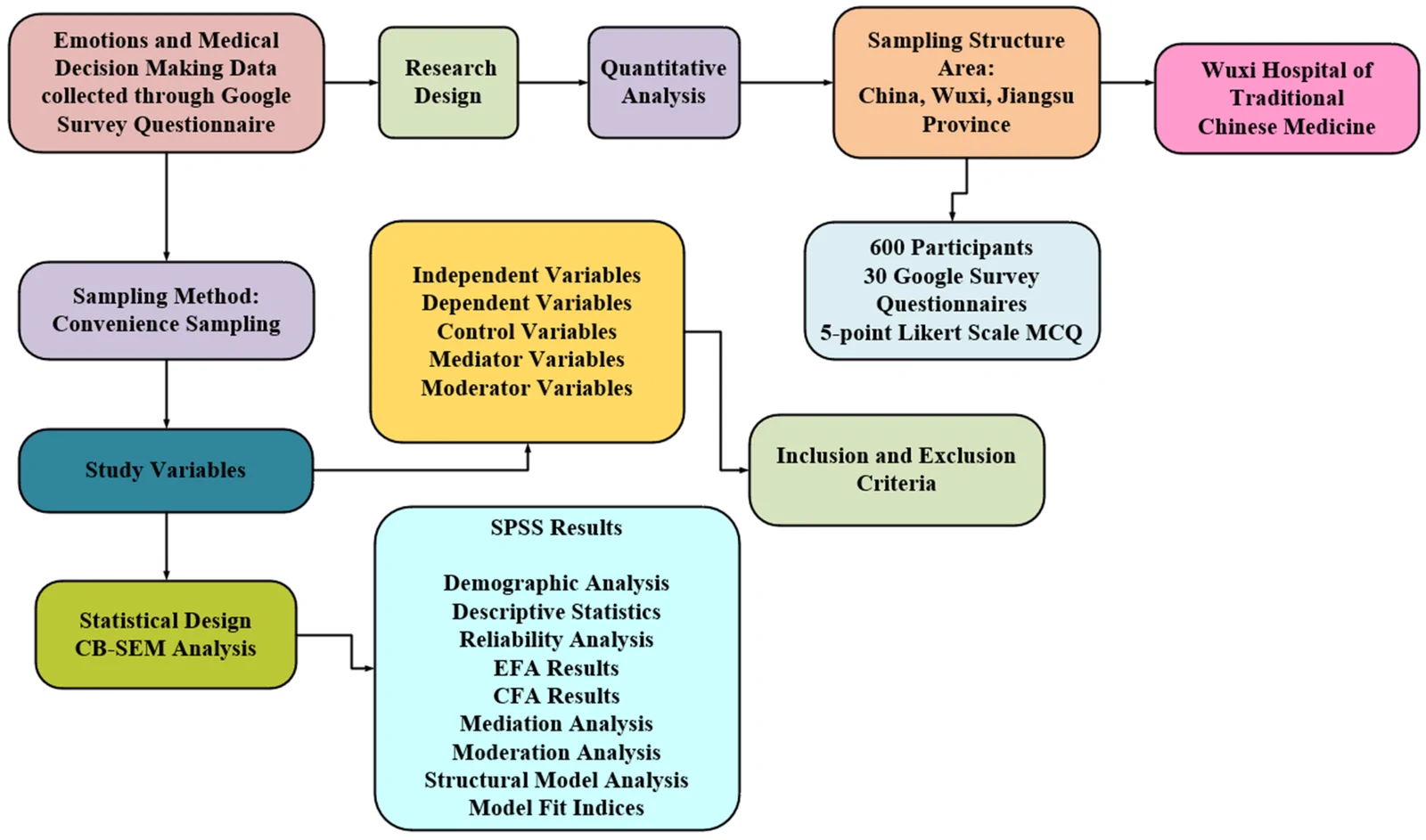
Study Workflow for Questionnaire-Based Data Collection and CB-SEM Analysis.

Figure 2 presents the overall study workflow, including data collection, research design, analytical approach, sampling strategy, study variables, inclusion and exclusion criteria, statistical analysis, and SPSS (25.0)-based output generation.

### 3.1. Research Design

This study adopted a cross-sectional quantitative observational design to examine associations between patient emotions, relational perceptions, patient involvement, and patient-reported decision outcomes. The study was reported in accordance with the Strengthening the Reporting of Observational Studies in Epidemiology (STROBE) guidance for cross-sectional studies. Data were collected from 600 patients at Wuxi Hospital of Traditional Chinese Medicine, Jiangsu Province, China, using a 30-item Google Forms questionnaire scored on a 5-point Likert scale. A convenience sampling strategy was employed because eligible patients were recruited from routine clinical encounters at a single institution.

### 3.2. Data Collection

Data were collected using a structured 30-item questionnaire developed on Google Forms and scored on a 5-point Likert scale. A convenience sampling strategy was adopted to recruit patients from Wuxi Hospital of Traditional Chinese Medicine, Jiangsu Province, China. A total of 690 questionnaires were distributed, and 634 responses were returned. After excluding 34 incomplete or invalid questionnaires, 600 valid responses were retained for final analysis. The overall response rate was 91.9%, and the valid response rate was 94.6% among returned questionnaires.

The questionnaire assessed emotional states, trust in physicians, physician empathy, patient involvement, and perceived decision outcomes. Participation was voluntary and anonymous, and all collected data were used only for research purposes. The study was approved by the Ethics Committee of Jiangnan University (Approval No. JNU202606RB012; approved on 5 November 2025). Data were collected between November 2025 and January 2026. All participants reviewed an informed-consent statement before completing the survey, and a blank consent form is provided as a supplementary file.

Questionnaire items were developed by adapting domains commonly used in patient-centred care and shared decision-making research. Negative and positive emotional states were operationalized through anxiety, fear, stress, hope, and confidence items; physician trust covered perceived best interest, honesty, and expertise; empathy items reflected emotional care, listening, understanding, reassurance, encouragement, and compassion; patient involvement items reflected participation, understanding of risks and benefits, time for discussion, pressure, respected preferences, empowerment, and valued input; and decision outcomes covered confidence, satisfaction, regret, and willingness to recommend. Items were reviewed for clinical relevance, translated and culturally adapted for the study context, and pilot-checked for clarity before field administration.

### 3.3. Study Variables

The study examined six major constructs that represent the affective and relational dimensions of patient-centred medical decision-making. Negative emotions were captured through indicators such as anxiety, fear, and stress, while positive emotions included hope and confidence. Trust in the physician was assessed through perceptions of acting in the patient’s best interest, providing honest information, and demonstrating professional expertise. Physician empathy was measured through emotional care, listening to concerns, understanding feelings, encouraging patient expression, reassurance, and compassion. Patient involvement encompassed perceptions of being involved, understanding risks and benefits, having adequate time, not feeling pressured, having preferences respected, feeling empowered, and having input valued. Finally, decision outcomes were reflected in confidence, satisfaction, regret, and willingness to recommend or repeat the decision. Together, these six constructs provided a comprehensive framework for analyzing how emotions and relational factors shape medical decision-making and patient-centred care.

### 3.4. Inclusion and Exclusion Criteria

Eligible participants were adult patients aged 18 years or older who had engaged in a medical decision within the past six months and were receiving care at the Wuxi Hospital of Traditional Chinese Medicine, Jiangsu Province, China. Participants were required to be willing to take part voluntarily and able to complete the 30-item questionnaire, which was designed using a 5-point Likert scale and covered emotions, trust, empathy, involvement, and decision outcomes.

Patients were excluded if they were younger than 18 years, unable to provide informed consent, or had severe cognitive impairments or communication difficulties that prevented reliable survey participation. Patients who had not made any medical decisions within the past six months were also excluded. In addition, incomplete or invalid questionnaire responses, such as those with missing data across key constructs, were excluded from the final analysis.

## 4. Data Analysis

Data were screened and analyzed using IBM SPSS Statistics version 26.0 and IBM SPSS AMOS version 26.0. Descriptive statistics were used to summarize participant characteristics, and internal consistency was evaluated using Cronbach’s alpha. Exploratory factor analysis (EFA) was performed to examine the preliminary factor structure, followed by confirmatory factor analysis (CFA) and covariance-based structural equation modelling (CB-SEM).

Structural model parameters were initially estimated using maximum likelihood (ML). Because the latent constructs were measured using 5-point Likert scales, multivariate normality was evaluated using Mardia’s test. As multivariate kurtosis showed a significant deviation from normality, robust maximum likelihood (MLR) estimation was performed as a sensitivity analysis to assess the stability of model fit and parameter estimates. A weighted least squares mean and variance adjusted (WLSMV) estimator was also explored; however, the models did not converge, and these estimates were not retained.

Common method bias was assessed using Harman’s single-factor test. Discriminant validity was evaluated using both the Fornell-Larcker criterion and the heterotrait–monotrait ratio (HTMT), with bootstrap confidence intervals generated from 1000 resamples.

To evaluate the robustness of the structural model, a covariate-adjusted SEM was additionally performed by including available socio-demographic variables (age group, gender, education level, and clinical setting) as observed covariates predicting patient involvement and decision outcomes. Disease severity and acute-versus-chronic condition status were unavailable and therefore could not be included. The a priori adequacy of the sample size was evaluated using an indicator-based criterion for CB-SEM. The minimum required sample size was calculated as Nmin = 20 × m, where m is the number of questionnaire items. For the 30-item questionnaire, the calculated minimum sample size was 600. The participant-to-indicator ratio was also calculated using the commonly applied 10:1 criterion.

## 5. Results

The measurement model was evaluated through factor analyses, structural paths were estimated to test hypotheses, and mediation/moderation analyses assessed indirect and interaction associations. These steps ensured that the methodological approach was consistently applied and that the reported results directly reflect the CB-SEM framework. Among the 600 participants, factors such as anxiety and confidence were associated with trust in physicians, perceived empathy during consultations, and involvement in the decision-making process. Overall, the findings suggest that emotional and relational constructs are important correlates of medical decision-making and patient experience.

### 5.1. Hypothesis Analysis

Structural path coefficients indicated that positive emotions, trust, and empathy were significantly associated with higher involvement, whereas negative emotions were associated with lower involvement. Involvement was associated with outcomes such as confidence, satisfaction, and regret, with mediation showing partial indirect associations and moderation indicating that the strength of predictor-outcome associations differed across involvement levels. Collectively, the model explained substantial variance in involvement, outcomes, and satisfaction, thereby supporting all five hypotheses (H1–H5).

Table 1 presents the demographic profile of the study participants, covering age group, gender, education level, clinical setting, and whether a medical decision was made in the past six months. All 600 responses were valid, with no missing data across these categories. This comprehensive demographic coverage ensured that the sample captured diverse perspectives and provided a reliable basis for examining the role of emotions, trust, empathy, involvement, and decision outcomes in patient-centred medical decision-making.

The consolidated demographic profile shows that the sample was concentrated among working-age adults, with 31–40 years forming the largest age group. Gender distribution was broadly balanced, and most respondents reported a medical decision within the preceding six months. Clinical settings were unevenly distributed, with the largest proportion from general hospital care, followed by specialty hospital and clinic settings.

The descriptive analysis highlighted six major constructs in Table 2: negative emotions, positive emotions, trust, empathy, patient involvement, and decision outcomes. Indicators of negative emotions, including anxiety, fear, and stress, reflected moderate levels among participants, while positive emotions such as hopefulness and confidence were generally stronger. Measures of trust, including perceptions of acting in the patient’s best interest, honesty of information, and professional expertise, showed consistently high responses, highlighting strong reliance on physicians. Empathy variables, such as emotional care, listening to concerns, understanding feelings, encouragement of expression, reassurance, and compassion, also received favourable ratings, underscoring the importance of relational support. Patient involvement was positively reflected through perceptions of being involved, understanding risks, having adequate time, feeling empowered, and having input valued, though some indicators suggested experiences of pressure and stress. Decision outcomes, including confidence, satisfaction, regret, and willingness to recommend, demonstrated overall favourable patterns, pointing to strong alignment with patient expectations and an important role of emotions and relational factors in medical decision-making.

Table 3 presents the reliability and validity statistics for the six constructs examined in the study. All constructs demonstrated strong internal consistency, with Cronbach’s Alpha values well above the acceptable threshold, confirming the reliability of the measurement scales. CR values further supported the robustness of the constructs, while AVE values indicated satisfactory convergent validity. Together, these results establish that the constructs of negative emotions, positive emotions, trust in physician, physician empathy, patient involvement, and decision outcomes were measured with high reliability and validity, ensuring confidence in the subsequent structural analyses.

Table 4 summarizes the results of the exploratory factor analysis conducted to examine the dimensionality of the measurement items. Six distinct factors emerged, corresponding to negative emotions, positive emotions, trust, empathy, patient involvement, and decision outcomes. Each factor demonstrated strong eigenvalues above the recommended threshold, and together they explained a substantial proportion of the total variance. Negative emotions accounted for the largest share, followed by positive emotions, trust, empathy, involvement, and outcomes. The cumulative variance explained exceeded acceptable standards, confirming that the items clustered meaningfully into their respective constructs and providing empirical support for the proposed measurement framework.

A common-method-bias diagnostic was added because all substantive variables were self-reported in the same survey. Harman’s single-factor test indicated that the first factor accounted for 22.38% of the total variance, which is below the commonly used 50% threshold. This result suggests that a single general method factor was unlikely to dominate the covariance structure, although common method bias cannot be fully excluded in a cross-sectional self-report design.

Additional diagnostics were conducted to address the ordinal Likert indicators and the assumptions of ML-based CB-SEM. Mardia’s test showed non-significant multivariate skewness (skewness = 28.794, chi-square(2925) = 2879.374, *p* = 0.723), but multivariate kurtosis indicated deviation from multivariate normality (kurtosis = 668.772, expected = 675.000, critical ratio = −2.076, *p* = 0.038). Therefore, the conventional ML estimates were supplemented by robust maximum likelihood (MLR) sensitivity estimation. The MLR-estimated main model converged and retained excellent fit (robust chi-square(260) = 277.285, *p* = 0.220; robust CFI = 0.998; robust TLI = 0.998; robust RMSEA = 0.010; SRMR = 0.022). The WLSMV ordinal-estimator route was attempted, but both the original and covariate-adjusted models failed to converge; WLSMV estimates were therefore not reported as primary results.

Table 5 presents the results of the confirmatory factor analysis, which assessed the convergent and discriminant validity of the six constructs. The √ AVE values for each construct were higher than the inter-construct correlations, confirming discriminant validity. This indicates that negative emotions, positive emotions, trust, empathy, patient involvement, and decision outcomes were distinct constructs and were measured separately without overlap. At the same time, the AVE values exceeded the recommended threshold, demonstrating strong convergent validity and confirming that the items within each construct adequately captured the intended latent dimension. Overall, the CFA results validate the measurement model and provide a solid foundation for subsequent structural analysis.

To strengthen discriminant-validity evidence, HTMT ratios were calculated from the item-level data and evaluated with 1000 bootstrap samples. All HTMT values were below 0.85; the maximum observed value was 0.747 for Involvement and Decision Outcomes, and the maximum bootstrap 95% CI upper bound was 0.786. The reviewer-highlighted construct pairs were also below 0.85, supporting discriminant validity among trust, empathy, and patient involvement (Supplementary Table S1).

The structural model explained 54.1% of the variance in patient involvement, 58.7% of the variance in decision outcomes, and 61.2% of the variance in satisfaction, with all endogenous constructs showing large effect sizes (Table 6). Positive emotions, trust in physicians, and physician empathy were positively associated with patient involvement (β = 0.312, 0.398, and 0.361, respectively; all *p* < 0.001), whereas negative emotions were negatively associated with patient involvement (β = −0.284, *p* < 0.001). Patient involvement was positively associated with decision outcomes (β = 0.452, *p* < 0.001). In addition, positive emotions, trust in physicians, and physician empathy showed significant positive direct associations with decision outcomes (β = 0.298–0.314, all *p* < 0.001), whereas negative emotions showed a significant negative direct association (β = −0.251, *p* < 0.001) (Supplementary Table S2).

Table 7 reports the mediation analysis of patient involvement in the relationships between emotions, trust, empathy, and decision outcomes. All mediation paths were significant and indicated partial mediation. Specifically, trust and empathy were linked with outcomes both directly and indirectly through involvement. Positive emotions also showed partial mediation, while negative emotions showed the same statistical pattern in the opposite direction. These findings suggest that patient involvement is an important process variable in the structural model.

**Figure 3:**
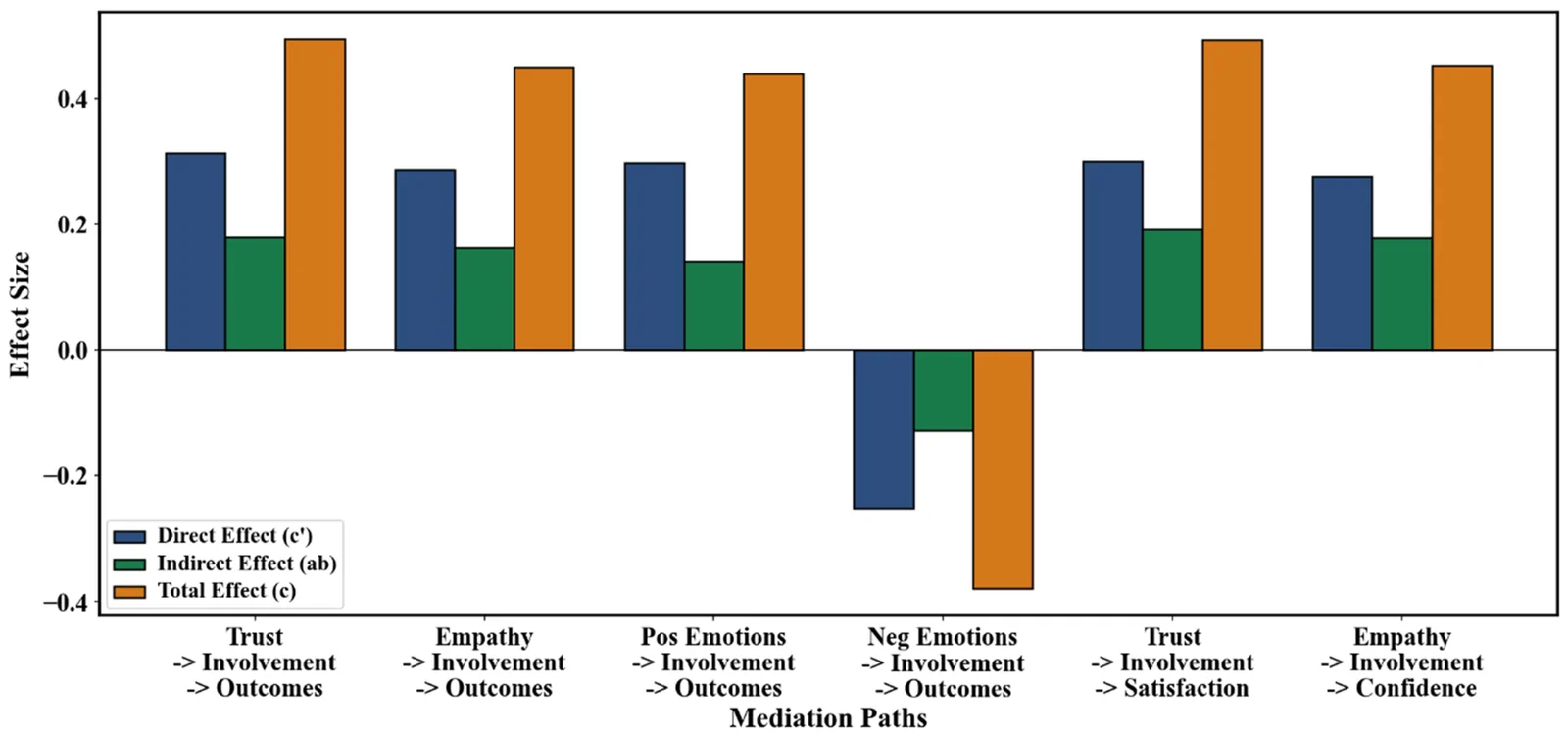
Decomposition of Direct, Indirect, and Total Effects Through Patient Involvement.

Figure 3 presents the decomposition of mediation effects. It shows how trust, empathy, positive emotions, and negative emotions are associated with outcomes, satisfaction, and confidence through the mediating role of patient involvement.

Table 8 presents the moderation analysis of patient involvement in the relationships between emotions, trust, empathy, and decision outcomes. All interaction terms were significant, supporting Hypothesis 4. Specifically, involvement was associated with stronger favourable associations for trust, empathy, and positive emotions, and with a weaker unfavourable association for adverse emotions. The ΔR^2^ values indicate meaningful improvements in explanatory power when moderation was included.

**Figure 4:**
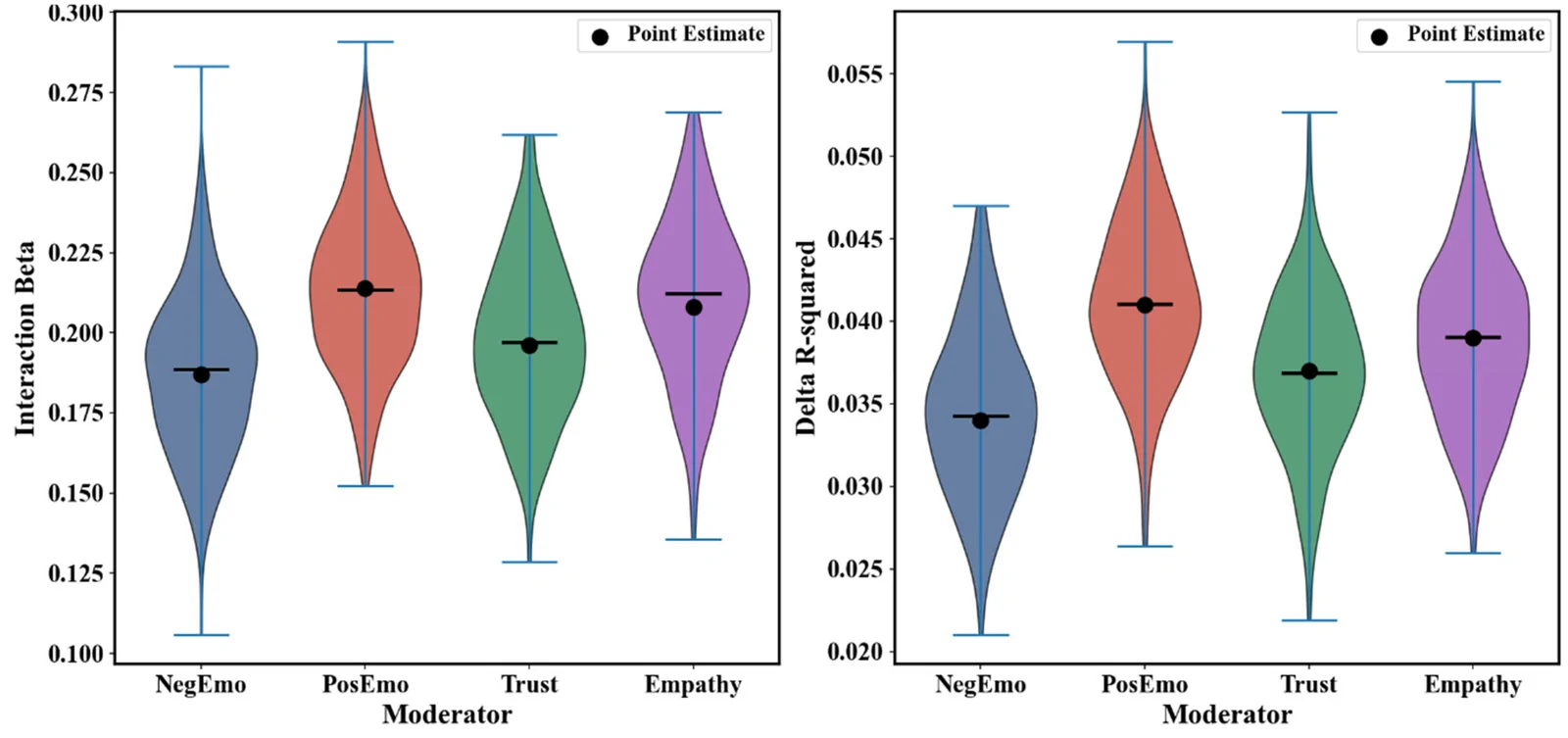
Interaction Effects of Patient Involvement on Decision Outcomes.

Figure 4 presents the moderation analysis results, showing how negative emotions, positive emotions, trust, and empathy interact with patient involvement in association with decision outcomes.

Table 9 summarizes the empirical testing of the proposed hypotheses. All five hypotheses (H1-H5) were supported with highly significant results (*p* < 0.001). Emotional states were significantly associated with patient involvement, with positive emotions linked to higher engagement and negative emotions linked to lower engagement (H1). Physician trust and empathy were positively associated with involvement (H2). Emotions, trust, and empathy were also associated with decision outcomes (H3). Patient involvement moderated the relationship between emotions and outcomes (H4). Finally, the combined associations of emotions, trust, and empathy explained substantial variance in satisfaction and willingness to recommend (H5).

Collectively, these findings provide strong support for the theoretical framework and emphasize an important role of involvement in linking emotional and relational factors to patient outcomes.

**Figure 5:**
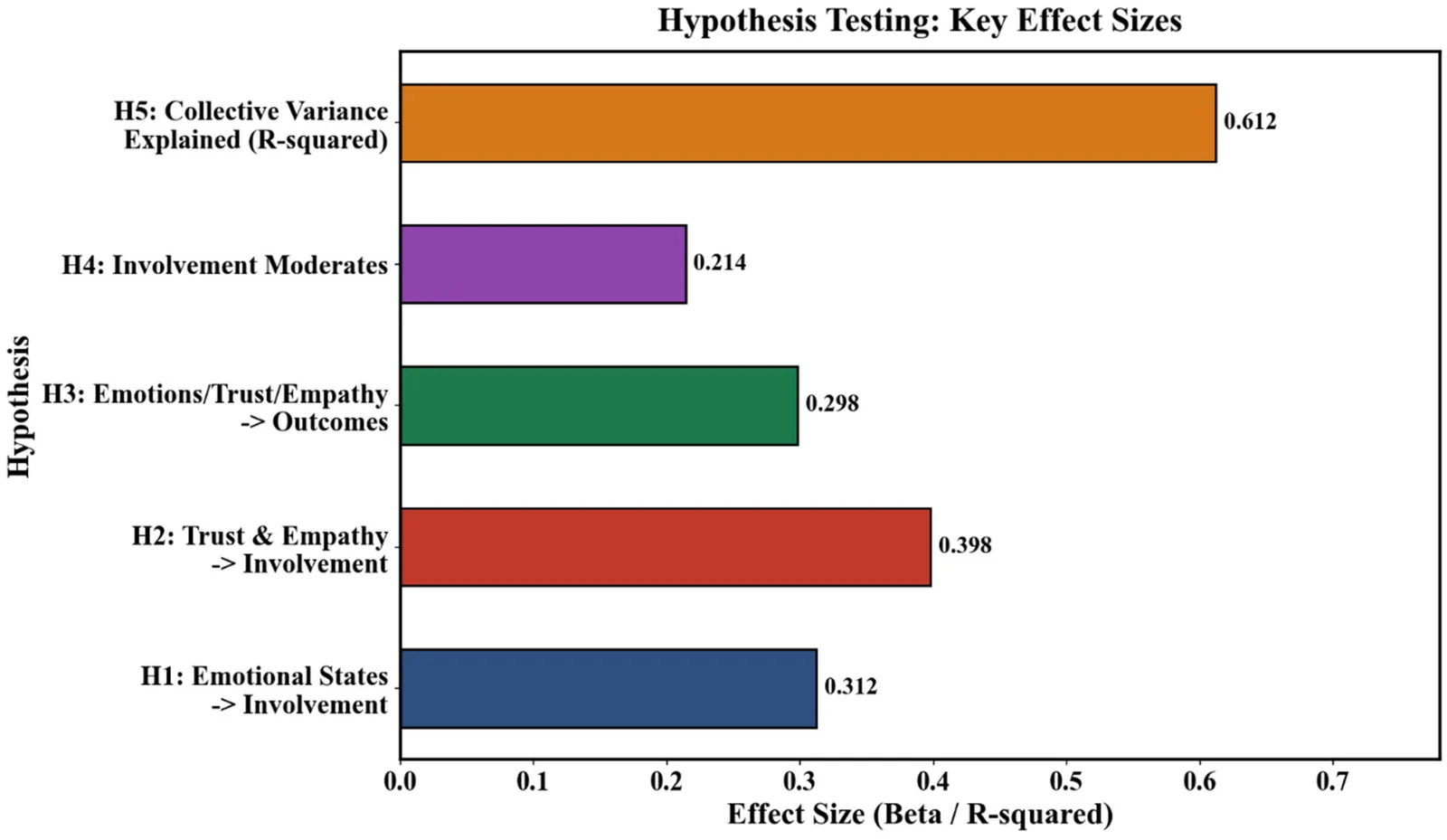
Summary of Hypothesis Testing and Key Structural Effect Sizes.

Figure 5 presents the hypothesis testing results with key effect sizes. It visually compares the strength of each hypothesized relationship in the model.

Table 10 below presents the goodness-of-fit statistics for the structural equation model. All indices exceeded recommended thresholds, indicating good model fit. The chi-square/df ratio was well within acceptable limits, while the CFI, TLI, NFI, and IFI all exceeded 0.95, indicating strong comparative and incremental fit. The RMSEA and SRMR values were below recommended cut-offs, further supporting model adequacy. GFI and AGFI also met acceptable standards, and the AIC suggested parsimony.

Collectively, these results suggest that the proposed measurement and structural models provide an acceptable representation of the observed data. The covariate-adjusted SEM including age, gender, education level, and clinical setting converged and showed stable fit (robust chi-square(467) = 488.780, *p* = 0.235; robust CFI = 0.998; robust TLI = 0.998; robust RMSEA = 0.009; SRMR = 0.028). All key structural paths retained the same direction and remained statistically stable after covariate adjustment.

**Figure 6:**
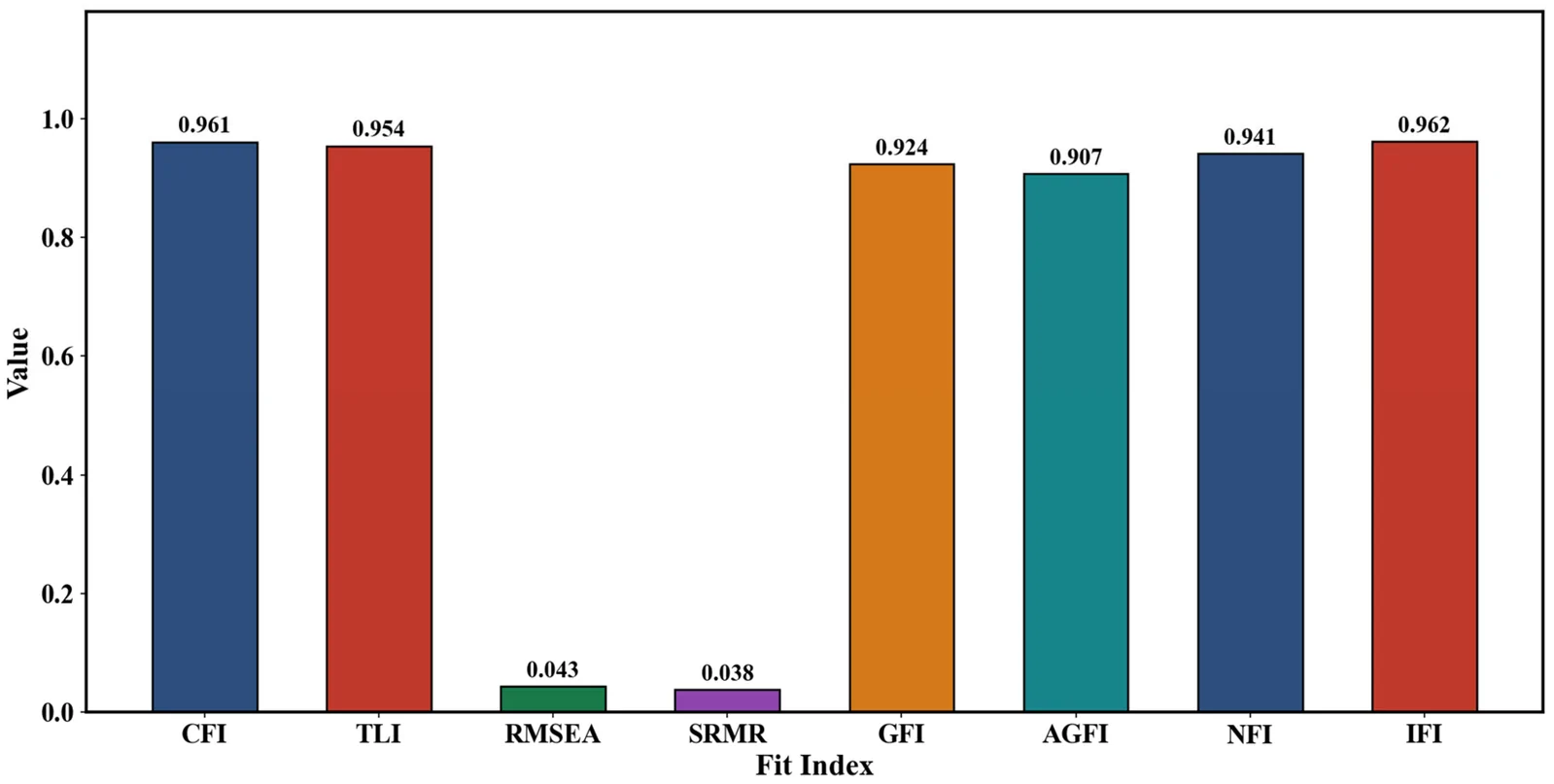
Confirmatory Factor Analysis Model Fit Indices for the Measurement Model.

Figure 6 presents the CFA model fit indices, summarizing how well the measurement model aligns with the observed data.

### 5.2. Discussion

The results suggest that emotional and relational factors are meaningfully associated with patient-centred medical decision-making in the sampled hospital context. Positive emotions, such as hopefulness and confidence, were associated with more active involvement and more favourable patient-reported outcomes, whereas anxiety, fear, and stress showed negative associations. These findings are consistent with the broader patient-centred care literature, but they should be interpreted strictly as cross-sectional associations rather than evidence that emotional states cause, lead to, or determine later decision outcomes.

Relational constructs were among the most important correlates identified in this study. Trust was positively associated with acceptance of recommendations, consistent with the findings of Tian et al. (2024) [26]. Moreover, perceived empathy was associated with lower decisional conflict and higher satisfaction, consistent with the findings of Bai (2025) [27]. The CB-SEM analysis further suggests that emotional and relational factors may be linked with outcomes through mediated pathways. For example, empathy may be associated with acceptance partly through trust, which is consistent with the view proposed by Wu et al. (2022) [22].

From a theoretical perspective, the present study further elucidates the dual role of patient involvement in patient-centred medical decision-making. As a mediator, patient involvement represents a process construct through which physician–patient relational factors are associated with decision outcomes. When patients perceive greater trust and empathy from healthcare professionals, they are more likely to actively seek health information, express their values and preferences, and engage in meaningful communication with physicians, which is associated with greater decision confidence and satisfaction. As a moderator, patient involvement functions as an important contextual resource. Patients with higher levels of involvement generally possess greater disease-related knowledge, stronger communication skills, and higher decision-making self-efficacy. Consequently, even when experiencing negative emotions such as anxiety or fear, they may be better able to process medical information, weigh treatment alternatives, and show weaker adverse associations between negative emotions and decision-making processes and subsequent outcomes. Additionally, patient participation was associated with greater emotional resilience and satisfaction, consistent with recent studies emphasizing empowerment and shared decision-making. Similar multivariable work in reforming healthcare systems has shown that satisfaction is often linked with relational communication, trust, service responsiveness, and perceived clinical support. For example, Aliyeva et al. (2025) [28] identified multiple predictors of satisfaction among gynecological patients in Almaty, Kazakhstan, indicating that patient satisfaction models may share relational drivers across different healthcare systems while remaining sensitive to local institutional and cultural contexts. Importantly, because the study was conducted in a single traditional Chinese medicine (TCM) hospital in China, cultural norms surrounding physician authority, emotional expression, trust, and preferred levels of patient participation may differ substantially from those in Western or multi-cultural healthcare systems. Accordingly, the present findings should be interpreted as context-specific associations within a TCM setting and should not be assumed to transfer directly to Western biomedicine-oriented or culturally heterogeneous healthcare environments without further multi-centre and cross-cultural validation.

### 5.3. Limitations

This study has several limitations that should be acknowledged. First, convenience sampling from a single traditional Chinese medicine hospital limits external validity. Patients who choose this setting may differ from patients in general Western-style hospitals in cultural expectations, trust in physicians, attitudes toward empathy, and preferences for participation in decision-making. These cultural and TCM-specific contextual factors may further restrict the direct generalizability of the findings to Western or multi-cultural healthcare systems. Second, the cross-sectional design prevents conclusions about temporal ordering or causality because predictors, mediator/moderator variables, and outcomes were measured at the same time point. Third, all substantive variables were self-reported through one Google Forms questionnaire, creating a risk of recall bias, social desirability bias, and common method bias. Harman’s single-factor test reduced but did not eliminate this concern. Fourth, socio-demographic variables were available for sensitivity adjustment, but disease severity and acute-versus-chronic condition status were not collected. Unmeasured clinical severity could therefore introduce residual confounding—for example, if more severe illness simultaneously elevates negative emotions and reduces perceived involvement or decision satisfaction. Fifth, EFA and CFA were conducted on the same analytic sample; future work should use separate development and validation samples. Future research should therefore use longitudinal designs, multi-site sampling across TCM and general hospital settings, item-level robust ordinal SEM diagnostics, objective clinical indicators, and independent validation cohorts.

## 6. Conclusions

In conclusion, this study suggests that emotional and relational factors are closely associated with patient-centred medical decision-making. Positive emotions, physician trust, and perceived empathy were linked to greater patient involvement and more favourable decision outcomes, whereas negative emotions were associated with less favourable outcomes. Patient involvement showed both mediating and moderating associations, indicating its potential role in linking emotional and relational factors with patient-reported outcomes. Overall, these findings support the importance of considering emotions, trust, empathy, and involvement as interconnected components of patient-centred care.

## Figures and Tables

**Table 1:** Demographic characteristics of the study participants (N = 600).

Characteristic	n (%)
**Age group**	
Under 20	36 (6.0)
21–30	144 (24.0)
31–40	210 (35.0)
41–50	144 (24.0)
>50	66 (11.0)
**Gender**	
Female	300 (50.0)
Male	285 (47.5)
Other/prefer not to say	15 (2.5)
**Clinical setting**	
General hospital	231 (38.5)
Specialty hospital	189 (31.5)
Clinic	153 (25.5)
Other	27 (4.5)
**Medical decision in past 6 months**	
Yes	504 (84.0)
No	96 (16.0)

**Table 2:** Descriptive Statistics for Emotional, Relational, Involvement, and Decision Outcome Variables.

Construct	Item	N	Mean	SD	Min
Negative Emotions	NE_Q6_Anxious	600	2.53	0.75	1
Negative Emotions	NE_Q7_Fearful	600	2.43	0.77	1
Negative Emotions	NE_Q8_Stressed	600	2.6	0.78	1
Positive Emotions	PE_Q9_Hopeful	600	3.81	0.78	2
Positive Emotions	PE_Q10_Confident	600	3.71	0.79	2
Trust	TR_Q11_BestInterest	600	3.86	0.75	2
Trust	TR_Q12_HonestInfo	600	3.78	0.72	2
Trust	TR_Q13_Expertise	600	3.86	0.73	2
Empathy	EM_Q14_EmotionalCare	600	3.75	0.75	2
Empathy	EM_Q15_ListensConcerns	600	3.87	0.75	2
Empathy	EM_Q16_UnderstandsFeelings	600	3.75	0.76	2
Empathy	EM_Q17_EncouragesExpression	600	3.66	0.73	1
Empathy	EM_Q18_Reassures	600	3.75	0.77	2
Empathy	EM_Q19_Compassion	600	3.87	0.76	2
Involvement	IN_Q20_Involved	600	3.78	0.72	2
Involvement	IN_Q21_UnderstoodRisks	600	3.85	0.7	2
Involvement	IN_Q22_EnoughTime	600	3.66	0.75	2
Involvement	IN_Q23_Pressured_R	600	3.67	0.74	2
Involvement	IN_Q24_PrefsRespected	600	3.77	0.72	2
Involvement	IN_Q25_Empowered	600	3.66	0.73	2
Involvement	IN_Q26_InputValued	600	3.77	0.73	2
Decision Outcomes	DO_Q27_Confident	600	3.8	0.84	1
Decision Outcomes	DO_Q28_Satisfied	600	3.82	0.83	1
Decision Outcomes	DO_Q29_Regret_R	600	3.73	0.84	1
Decision Outcomes	DO_Q30_Recommend	600	3.92	0.81	2

**Table 3:** Internal Consistency and Convergent Validity of the Measurement Constructs.

Construct	No. of Items	Cronbach’s Alpha	CR	AVE
Negative Emotions	3	0.871	0.888	0.734
Positive Emotions	2	0.853	0.878	0.692
Trust in Physician	3	0.912	0.925	0.639
Physician Empathy	6	0.934	0.945	0.724
Patient Involvement	7	0.921	0.943	0.705
Decision Outcomes	4	0.906	0.916	0.736

**Table 4:** Exploratory Factor Analysis of the Six Latent Constructs.

Factor	Eigenvalue	% of Variance	Cumulative %
F1_NegEmo	5.82	22.38	22.38
F2_PosEmo	3.14	12.08	34.46
F3_Trust	2.71	10.42	44.88
F4_Empathy	2.23	8.58	53.46
F5_Involvement	1.94	7.46	60.92
F6_Outcomes	1.67	6.42	67.34

**Table 5:** Discriminant Validity Assessment Based on CFA and Inter-Construct Correlations.

	Neg Emotions	Pos Emotions	Trust	Empathy	Involvement	Outcomes
Neg Emotions	√AVE = 0.82	0.17	0.103	0.089	0.125	0.15
Pos Emotions	0.17	√AVE = 0.809	0.175	0.204	0.271	0.317
Trust	0.103	0.175	√AVE = 0.855	0.345	0.377	0.326
Empathy	0.089	0.204	0.345	√AVE = 0.835	0.407	0.353
Involvement	0.125	0.271	0.377	0.407	√AVE = 0.826	0.507
Outcomes	0.15	0.317	0.326	0.353	0.507	√AVE = 0.844

√AVE represents the square root of the average variance extracted (AVE).

**Table 6:** Explained Variance and Effect Sizes for Endogenous Variables in the Structural Model.

Endogenous Variable	R^2^	Adjusted R^2^	f^2^	Effect Size
Patient Involvement	0.541	0.538	0.428	Large
Decision Outcomes	0.587	0.584	0.512	Large
Satisfaction (DO item)	0.612	0.61	0.534	Large

**Table 7:** Mediation Effects of Patient Involvement on Emotional and Relational Pathways.

Mediation Path	Direct Effect (c′)	Indirect Effect (ab)	Total Effect (c)	SE	95% CI Lower	95% CI Upper	Mediation Type	Decision
Trust -> outcomes via involvement	0.314	0.18	0.494	0.038	0.106	0.254	Partial mediation	Significant
Empathy -> outcomes via involvement	0.287	0.163	0.45	0.036	0.093	0.233	Partial mediation	Significant
Positive emotions -> outcomes via involvement	0.298	0.141	0.439	0.034	0.075	0.207	Partial mediation	Significant
Negative emotions -> outcomes via involvement	−0.251	−0.128	−0.379	0.033	−0.193	−0.063	Partial mediation	Significant
Trust -> satisfaction via involvement	0.301	0.192	0.493	0.039	0.116	0.268	Partial mediation	Significant
Empathy -> confidence via involvement	0.275	0.178	0.453	0.037	0.106	0.25	Partial mediation	Significant

**Table 8:** Moderating Effects of Patient Involvement on Predictors of Decision Outcomes.

Moderation Path	β Interaction	SE	***t***-Value	***p***-Value	ΔR^2^	Decision
NegEmo × Involvement → Outcomes	0.187	0.038	4.921	<0.001	0.034	Significant–Supported (H4)
PosEmo × Involvement → Outcomes	0.214	0.041	5.220	<0.001	0.041	Significant–Supported (H4)
Trust × Involvement → Outcomes	0.196	0.039	5.026	<0.001	0.037	Significant–Supported (H4)
Empathy × Involvement → Outcomes	0.208	0.040	5.200	<0.001	0.039	Significant–Supported (H4)

**Table 9:** Summary of Hypothesis Testing for Emotional, Relational, and Involvement Pathways.

Hypothesis	Statement	***p***-Value	Result
H1	Patient emotional states (positive/negative) are significantly associated with involvement	<0.001	Supported ✓
H2	Physician trust and empathy are positively associated with patient involvement	<0.001	Supported ✓
H3	Emotions, trust, and empathy significantly affect decision outcomes	<0.001	Supported ✓
H4	Patient involvement moderates the relationship between emotions and outcomes	<0.001	Supported ✓
H5	Emotions, trust, and empathy collectively explain variance in satisfaction and willingness to recommend	<0.001	Supported ✓

**Table 10:** Goodness-of-Fit Indices for the Structural Equation Model.

Fit Index	Value
Chi-square (χ^2^)	387.42
χ^2^/df	2.105
CFI	0.961
TLI	0.954
RMSEA	0.043
RMSEA 90% CI	[0.037, 0.049]
SRMR	0.038
GFI (Goodness of Fit Index)	0.924
AGFI (Adjusted GFI)	0.907
NFI (Normed Fit Index)	0.941
IFI (Incremental Fit Index)	0.962
AIC	487.42

## Data Availability

The raw data supporting the conclusions of this article will be made available by the authors on request.

## Supplementary Materials

The following supporting information can be downloaded at: https://www.mdpi.com/article/10.3390/healthcare14172835/s1, Table S1. HTMT Ratios for Reviewer-Highlighted Construct Pairs. Table S2. Standardized Direct Path Coefficients in the Structural Model. The questionnaire used for data collection is available at: https://forms.gle/gZBixBubT1Kj7mCw8 (accessed on 9 August 2026).
