# Supplementary material for: Exploring the Interplay Between Emotions and Medical Decision-Making: Insights from an Empirical Patient-Centred Study
Source: Healthcare (Basel). 2026 Sep 3;14(17):2835. doi: 10.3390/healthcare14172835 (PMC13565710; doi:10.3390/healthcare14172835)
Supplement: Supplementary file 1 [file healthcare-14-02835-s001.zip › healthcare-4356597-supplementary.pdf]

Supplementary Materials

Supplementary Table S1. HTMT Ratios for Reviewer-Highlighted Construct Pairs.

Construct pair	HTMT	Bootstrap 95% CI	Judgment
Trust–Empathy	0.418	0.339 to 0.489	Below 0.85, supporting discriminant validity
Trust–Involvement	0.554	0.494 to 0.617	Below 0.85, supporting discriminant validity
Empathy–Involvement	0.590	0.522 to 0.646	Below 0.85, supporting discriminant validity

Supplementary Table S2. Standardized Direct Path Coefficients in the Structural Model.

Path	Standardized β	p-value	Interpretation
Positive emotions -> Patient involvement	0.312	<0.001	Positive association
Negative emotions -> Patient involvement	-0.284	<0.001	Negative association
Trust in physician -> Patient involvement	0.398	<0.001	Positive association
Physician empathy -> Patient involvement	0.361	<0.001	Positive association
Patient involvement -> Decision outcomes	0.452	<0.001	Positive association
Positive emotions -> Decision outcomes	0.298	<0.001	Positive direct association
Negative emotions -> Decision outcomes	-0.251	<0.001	Negative direct association
Trust in physician -> Decision outcomes	0.314	<0.001	Positive direct association
Physician empathy -> Decision outcomes	0.287	<0.001	Positive direct association
